# Assessment of Meteorological Variables and Air Pollution Affecting COVID-19 Cases in Urban Agglomerations: Evidence from China

**DOI:** 10.3390/ijerph19010531

**Published:** 2022-01-04

**Authors:** Mingyue Zhao, Yuanxin Liu, Amatus Gyilbag

**Affiliations:** 1 Institute of Environment and Sustainable Development in Agriculture, Chinese Academy of Agricultural Sciences, Beijing 100081, China; zhaomingyue@caas.cn (M.Z.); amatusmike@yahoo.com (A.G.); 2 Academy for Multidisciplinary Studies, Capital Normal University, Beijing 100048, China

**Keywords:** meteorological variables, air pollution, COVID-19, urban agglomeration, GAM

## Abstract

The 2019 novel coronavirus disease (COVID-19) has become a severe public health and social problem worldwide. A limitation of the existing literature is that multiple environmental variables have not been frequently elaborated, which is why the overall effect of the environment on COVID-19 has not been conclusive. In this study, we used generalized additive model (GAM) to detect the relationship between meteorological and air pollution variables and COVID-19 in four urban agglomerations in China and made comparisons among the urban agglomerations. The four urban agglomerations are Beijing-Tianjin-Hebei (BTH), middle reaches of the Yangtze River (MYR), Yangtze River Delta (YRD), and the Pearl River Delta (PRD). The daily rates of average precipitation, temperature, relative humidity, sunshine duration, and atmospheric pressure were selected as meteorological variables. The PM_2.5_, PM_10_, sulfur dioxide (SO_2_), nitrogen dioxide (NO_2_), ozone (O_3_), and carbon monoxide (CO) contents were selected as air pollution variables. The results indicated that meteorological and air pollution variables tended to be significantly correlated. Moreover, the nature of the relationship between severe acute respiratory syndrome coronavirus 2 (SARS-CoV-2) and meteorological and air pollution variables (i.e., linear or nonlinear) varied with urban agglomerations. Among the variance explained by GAMs, BTH had the highest value (75.4%), while MYR had the lowest value (35.2%). The values of the YRD and PRD were between the above two, namely 45.6% and 62.2%, respectively. The findings showed that the association between SARS-CoV-2 and meteorological and air pollution variables varied in regions, making it difficult to obtain a relationship that is applicable to every region. Moreover, this study enriches our understanding of SARS-CoV-2. It is required to create awareness within the government that anti-COVID-19 measures should be adapted to the local meteorological and air pollution conditions.

## 1. Introduction

The 2019 novel coronavirus disease (COVID-19) is an infectious disease caused by severe acute respiratory syndrome coronavirus 2 (SARS-CoV-2) [1,2], and the pandemic declared by the World Health Organization (WHO) is still an ongoing outbreak globally [3]. SARS-CoV-2 is highly transmissible and more than 258.85 million people have been infected, with more than 5 million reported deaths worldwide (https://voice.baidu.com/act/newpneumonia/newpneumonia/?from=osari_aladin_banner#tab4, accessed on 24 October 2021). Controlling the spread of SARS-CoV-2 via appropriate government intervention is an effective method [4]. China adopted a series of measures, including locking down cities [5], extending the Spring Festival holiday, and delaying the start of schools [6]. Even though China has passed several waves of the pandemic, the association between meteorological and air pollution variables and SARS-CoV-2 remains unclear. From the first report to outbreak, the characteristics of SARS-CoV-2 in different regions need to be better understood to provide valuable scientific support for global epidemic control and response.

Meteorological variables may affect the survival of viruses and epidemic transmission affecting droplet stability in the environment [7]. Air pollutants are gases or particles dispersed in the air, with different chemical and physical structures, and with heterogeneous effects on air quality, airborne pollen, climate, and eventually human health [8,9]. Several studies have examined the combined effects of meteorological variables and ecological components such as air pollution on human health and mortality [10,11,12]. Scientists have observed that the spread of SARS-CoV-2 is related to meteorological variables and air pollution [13,14]. Roviello and Roviello [15] analyzed the impact of SARS-CoV-2 in different Italian regions and their findings confirmed their hypothesis on the relationship between the severity of the pandemic and certain environmental factors, such as air pollution and scarcity of nondeciduous vegetation. Xie and Zhu [16] found that the mean temperature of the last two weeks was positively associated with newly confirmed SARS-CoV-2 cases, and a 1 °C increase in the mean temperature of the last week was associated with an almost 5% increase in the daily confirmed cases. He, et al. [17] indicated that particulate matter with an aerodynamic diameter ≤10 μm (PM_10_) concentrations decreased by 25% during the lockdown time spanning a few weeks in Chinese cities. Another study reported that PM_2.5_ was reduced by 54% in the lockdown period during the same time of the previous year [18]. Furthermore, ecological research indicated that there were significant relationships between influenza-like illness risk and air particulate matter in Beijing, China [19]. A global study found that pollen, sometimes in synergy with humidity and temperature, explained, on average, 44% of the infection rate variability [20]. No single environmental factor is directly responsible for the outbreak of COVID-19. For this reason, there are some deficiencies in the current research. Studies on the impact of a single environmental factor on SARS-CoV-2 may have inconsistent results. In addition, in the existing literature, multifactorial designs of multiple environmental variables have not been frequently elaborated.

In analyzing the long- and short-term effects, a generalized additive model (GAM) with spline fitting has been proposed to study the relationship between SARS-CoV-2 cases and meteorological variables and air pollutants [6,8,10,16]. The GAM provides a flexible specification of response by defining the model in terms of smooth function as a replacement for the detailed parametric relationships on the covariates [10]. Based on previous studies, we conclude a hypothesis that meteorological variables and air pollution jointly affect SARS-CoV-2, and this effect is spatially heterogeneous. To be specific, the association between SARS-CoV-2 and variables is not immutable and may be linear or nonlinear.

Urban agglomerations are key areas for population gathering [21]. Owing to high-level medical facilities, these areas have received cases from surrounding areas, and are thus key areas for prevention, control, and research. Cities in urban agglomerations tend to have closer economic and social coordination, which will have an impact on medical resources. In the context of the global spread of SARS-CoV-2, it remains unclear how meteorological variables and air pollution are related to SARS-CoV-2 transmission in urban agglomerations.

In this study, a time-series analysis of the cumulative daily number of confirmed cases of SARS-CoV-2 based on GAM was conducted in China, aiming at (1) exploring changes in meteorological variables, air pollution variables, and SARS-CoV-2 cases in different urban agglomerations; (2) clarifying the types of relationships between single variable and SARS-CoV-2; (3) quantifying the combined effects of multiple variables on SARS-CoV-2 transmission; and (4) comparing the effects of meteorological and air pollution variables on the spread of SARS-CoV-2 in different urban agglomerations.

## 2. Materials and Methods

### 2.1. Study Design

To test our primary hypothesis that air pollution and meteorological variables together influence SARS-CoV-2 infection and that the effects may vary in different regions, we performed a GAM with a quasi-Poisson link function based on previous studies [22]. The entire study period was from the day of first patient diagnosis to 29 February, which is basically from the first cases to the end of the first wave in 2020. Based on previous studies and the convenience of data acquisition in the study area, representative air pollution indicators and meteorological variables were selected. The daily dates of average precipitation (PRE, mm), temperature (TEM, °C), relative humidity (RH, %), sunshine duration (SD, h), and atmospheric pressure (AP, hPa) were selected as meteorological variables. The PM_2.5_ (μg/m^3^), PM_10_ (μg/m^3^), sulfur dioxide (SO_2_, μg/m^3^), nitrogen dioxide (NO_2_, μg/m^3^), ozone (O_3_, μg/m^3^), and carbon monoxide (CO, μg/m^3^) contents were selected as air pollution variables. Particles resulting from combustion processes are generally less than 2.5 μm and 10 μm, which are defined as PM_2.5_ and PM_10_, respectively.

### 2.2. Study Area

**Figure 1:**
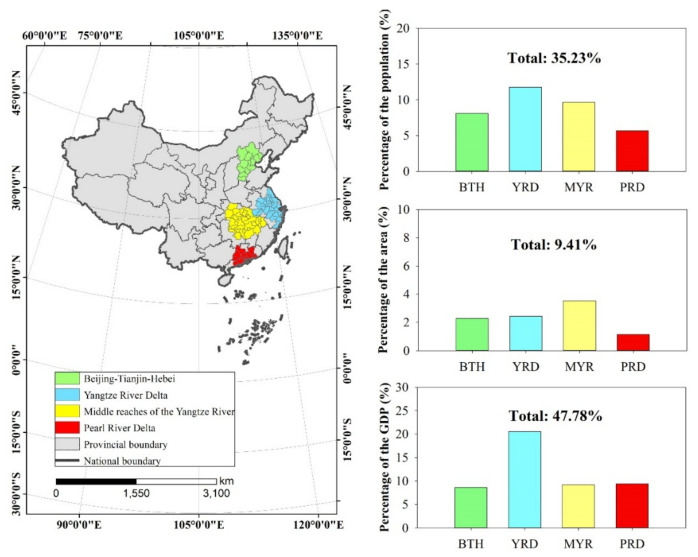
Four urban agglomeration locations in China and their populations, areas, and GDPs.

The middle reaches of the Yangtze River (MYR) are an urban agglomeration with the most confirmed cases, including Wuhan city, the center of the COVID-19 outbreak in China. As Wuhan is the worst-hit region and cases accounted for approximately 60% of cases in China, the comparison among Wuhan and other cities might be biased. Therefore, we omitted the cases in Wuhan in this study. In addition to MYR, there are three main urban agglomerations in China, named Beijing-Tianjin-Hebei (BTH), Yangtze River Delta (YRD), and Pearl River Delta (PRD) (Table S1). Four urban agglomeration locations in China and their populations, areas, and GDPs are shown in Figure 1. Given that daily confirmed SARS-CoV-2 cases are counted at a city level, cities without confirmed cases were excluded from our analysis of urban agglomerations. As of 2019, the population of the four urban agglomerations accounted for 35.23% of China’s population, of which the YRD had the largest population (165 million) (Figure 1). The four urban agglomerations accounted for 9.41% of the total land area, with the smallest being the PRD area (110,300 km^2^). In terms of gross domestic product (GDP), the total GDP of the four urban agglomerations accounted for 42.78% of the national GDP, among which the YRD contributed 20.35 trillion RMB.

### 2.3. Data

Daily SARS-CoV-2 confirmed case data were obtained from the National Health Commission of the People’s Republic of China, Beijing, China (http://www.nhc.gov.cn, accessed on 15 October 2020), and the study objective is cities in urban agglomerations of BTH, MYR, YRD, and PRD. A total of 34,034 data points from 85 cities were selected for analysis. After that, the cumulative daily number of confirmed cases of SARS-CoV-2 was calculated. The study period was from the day of first patient diagnosis to 29 February 2020, which was basically from the first cases to the end of the first wave.

Meteorological data were obtained from the National Meteorological Information Center, Beijing, China (http://data.cma.cn/, accessed on 12 March 2021), and air pollution data were downloaded from the China National Environmental Monitoring Centre, Beijing, China (http://www.cnemc.cn/, accessed on 12 March 2021). Social statistical data come from statistical yearbooks of provinces and cities.

### 2.4. Methods

Spearman’s rank correlation was used to investigate the association between two variables. The correlation coefficient was used to investigate the correlation between meteorological variables, air pollution, and SARS-CoV-2 cases. The GAM, as a semiparametric extension of the generalized linear model (GLM), helps to explore the nonlinear relationship between meteorological variables, air pollution, and SARS-CoV-2 cases [9,23]. GAM offers an open-ended solution in the case of considerable noise in the predictor variables [10]. The GAM was expressed as follows:

(1) $$g\left(\mu \left(Y\right)\right)=\beta_{0}\;+\;f_{1}\left(x_{1}\right)\;+\;f_{2}\left(x_{2}\right)\;+\ldots +\;f_{m}\left(x_{m}\right)$$

where g() is a link function; *μ*(*Y*) is the expectation of the response variable *Y*; *β*_0_ is the intercept; and *f*_i_(), *i* = 1, 2, …, m are the smoothing functions of predictor variables, with a specified parametric or nonparametric form.

The GAM allows a broad range of distributions for the response variable to be adopted and link functions for measuring the effects of the predictor variables on the dependent regressors [10]. Popularly used distributions in GAM modeling are normal, gamma, and Poisson distributions. Considering the distribution of variables and the characteristics of link functions, we choose the generalized additive quasi-Poisson model. Before GAM analysis, collinearity among the influencing variables of SARS-CoV-2 was first determined using the collinearity diagnosis function of SPSS (IBM SPSS Statistics 22). The results revealed that PM_2.5_ and PM_10_ had collinearity challenges and we removed PM_10_ from the GAM analysis. We judge the function form according to the value of degree of freedom in the GAM model hypothesis test results. When the degree of freedom is 1, the function is a linear equation, indicating that there is a linear relationship between the influencing factors and the response variables. When the degree of freedom is greater than 1, the expression function is a nonlinear curve equation, and there is a certain nonlinear relationship between the influencing factors and the response variables. When the degree of freedom is larger, the nonlinear relationship is more significant.

GAMs in our analysis were implemented via the “mgcv” package (version 1.8-28) of R software, Auckland, New Zealand (version 4.0.3). The statistical tests were two-sided, and *p* < 0.05 was considered statistically significant.

## 3. Results

### 3.1. Description of SARS-CoV-2 Daily Infection Cases and Meteorological and Air Pollution Variables

**Figure 2:**
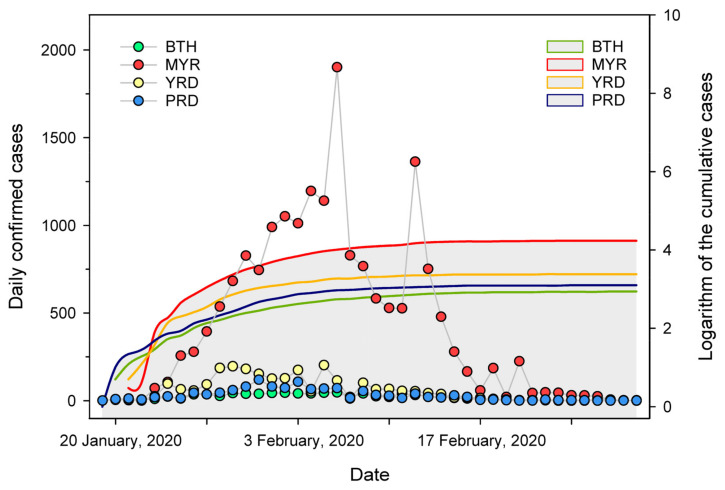
The daily confirmed cases and logarithm of the cumulative cases of SARS-CoV-2 in four urban agglomerations.

During the study period (from 18 January 2020 to 29 February 2020), the cumulative number of SARS-CoV-2 confirmed cases in China (including Hong Kong, Macao, and Taiwan Province) reached 79,824, including 50,340 in Wuhan city. The daily confirmed cases in four urban agglomerations and the logarithm of the cumulative cases are shown in Figure 2. In 2020, the daily confirmed cases of MYR increased rapidly from the beginning until reaching a peak on 6 February. Then, it fluctuated and declined. The number of daily confirmed cases was higher than that of BTH, YRD, and PRD in most cases. Before 17 February, the daily number of SARS-CoV-2 confirmed cases in the YRD was higher than that in BTH and PRD. The number of confirmed cases of YRD reached its maximum (202) on 5 February. From 24 January to 29 February, the logarithm of cumulative confirmed cases in MYR was consistently higher than in the other three agglomerations. After 6 February, this value tends to be stable with little fluctuation. Overall, the confirmed cases in MYR, BTH, YRD, and PRD were 69,930, accounting for 87% of the total cases in China. On 12 February, 15,152 newly confirmed cases in China were diagnosed (including 13,332 cumulative clinically diagnosed cases in Hubei). On 18 February, the daily number of newly cured and discharged coronavirus patients exceeded that of newly confirmed cases, and the number of confirmed cases began to drop. On 21 February, most provinces and equivalent administrative units in China started to downgrade their public health emergency response level in light of the local situation, and gradually lifted traffic restrictions.

During the study period, PRD had the highest temperature among the four urban agglomerations, while BTH had the lowest temperature (Figure S1). Heavy precipitation mainly occurred during 21–27 January, 3–8 February, and 10–17 February. In these three periods, YRD, MYR, and PRD had the highest cumulative precipitation. In terms of air pressure, YRD had the highest pressure most of the time, while BTH was relatively low. In addition, there was a negative correlation between sunshine duration and precipitation. During the study period, the relative humidity of PRD, YRD, and MYR was higher than that of BTH, which was consistent with the latitudinal relationship among them. The PM_2.5_ and SO_2_ in the air of BTH tended to be significantly higher than those of the other three urban agglomerations (Figure S2). There was an obvious downward trend for PM_2.5_ from 25 January to 10 February. The variation trend of PM_10_ is consistent with that of PM_2.5_, and the variation range is relatively large. PRD tended to have lower PM_2.5_ and SO_2_ contents, indicating better air quality. PRD had the highest ozone content in 45% of the study period, while BTH had the highest in 31% of the period. For NO_2_ and CO, another indicator of air pollution, BTH was highest for most of the period, followed by MYR, YRD, and PRD.

### 3.2. Correlation between SARS-CoV-2 Cases and Meteorological Variables and Air Pollution Variables

**Figure 3:**
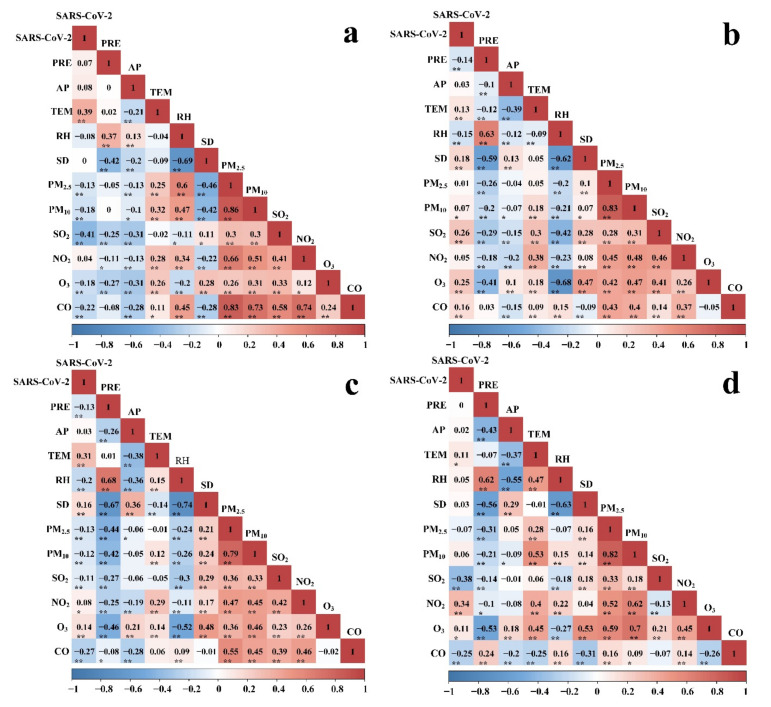
Spearman’s correlation between SARS-CoV-2 cases and meteorological variables and air pollution variables in BTH (**a**), MYR (**b**), YRD (**c**), and PRD (**d**). Notes: *, *p* < 0.05; **, *p* < 0.01.

Spearman’s correlation coefficients between the SARS-CoV-2 cases, meteorological variables, and air pollution are shown in Figure 3. In BTH, the SARS-CoV-2 cases were significantly positively correlated with TEM (0.39), but significantly negatively correlated with O_3_ (−0.18), SO_2_ (−0.41), CO (−0.22), PM_10_ (−0.18), and PM_2.5_ (−0.13) (Figure 3a). The results showed no significant correlation between SARS-CoV-2 and PRE, RH, AP, or SD. Among the other meteorological variables and air pollution variables, PM_2.5_ and PM_10_ had the highest positive correlation coefficient (0.86).

In the MYR, SARS-CoV-2 was positively correlated with SD (0.18), O_3_(0.25), SO_2_ (0.26), temperature (0.13), CO (0.16), and PM_10_ (0.07), and negatively correlated with RH (−0.15) and PRE (−0.14) (Figure 3b). In addition, PRE had no significant correlation with CO, but had a significant correlation with other variables (*p* < 0.01). PM_2.5_ and PM_10_ had the highest positive correlation coefficient (0.83). The positive correlation coefficient between O_3_ and SD reached 0.47.

In the YRD, SARS-CoV-2 was positively correlated with TEM (0.31), NO_2_ (0.08), SD (0.16), and O_3_ (0.14), but negatively correlated with PRE (−0.13), RH (−0.2), CO (−0.27), PM_2.5_ (−0.13), PM_10_ (−0.12), and SO_2_ (−0.11) (Figure 3c). PM_2.5_ and PM_10_ had the highest positive correlation coefficient (0.79). The lowest negative correlation coefficient was observed between PRE and SD (−0.67).

In the PRD, SARS-CoV-2 was positively correlated with NO_2_ (0.34), TEM (0.11), and O_3_ (0.11), but the correlation coefficients were small, while it was negatively correlated with CO (−0.25) and SO_2_ (−0.38) (Figure 3d). In addition, there was no significant correlation between SARS-CoV-2 and PM_10_, PM_2.5_, SD, AP, RH, or PRE. Among meteorological variables and air pollution variables, PM_2.5_ had a very significant correlation with PM_10_, and the correlation coefficient reached 0.82. At the same time, the negative correlation between RH and SD was very significant (−0.63).

### 3.3. The Response of SARS-CoV-2 to Meteorological and Air Pollution Variables

GAM was used to simulate the response of SARS-CoV-2 cumulative confirmed cases to meteorological and air pollution variables in the four urban agglomerations, and the results are shown in Table 1. In BTH, the deviance explanatory rate of meteorological and air pollution variables to the SARS-CoV-2 cases was 75.4%, and the significantly related factors were O_3_, AP, RH, SD, SO_2_, CO, and NO_2_. In the MYR, the deviance explained was 35.2%, which was the lowest value among the four urban agglomerations. The significantly related variables in MYR were PRE, O_3_, AP, PM_2.5_, SO_2_, CO, and NO_2_. In YRD, the deviance explained was 45.6%, and the significantly related factors were PRE, TEM, AP, RH, PM_2.5_, SO_2_, NO_2_, and CO. In PRD, the deviance explained was 62.2%, and the significantly related variables were O_3_, AP, RH, SD, PM_2.5_, SO_2_, CO, and NO_2_.

**Figure 4:**
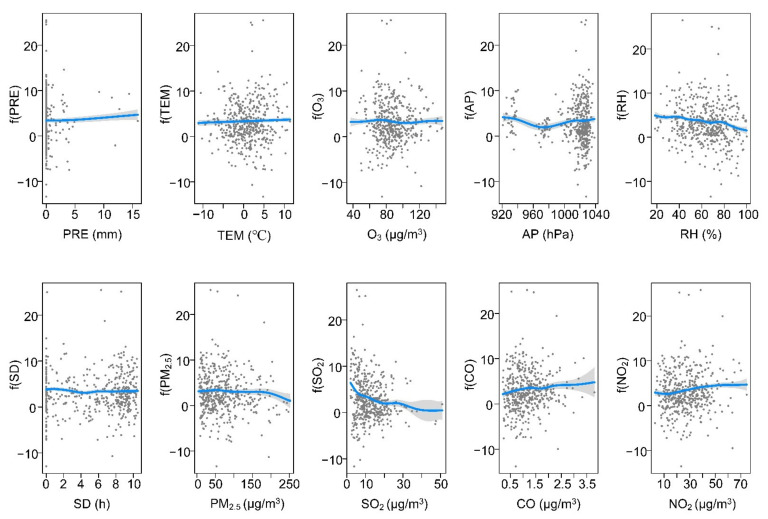
Exposure–response curves among meteorological variables, air pollution variables, and SARS-CoV-2 cases in BTH.

**Figure 5:**
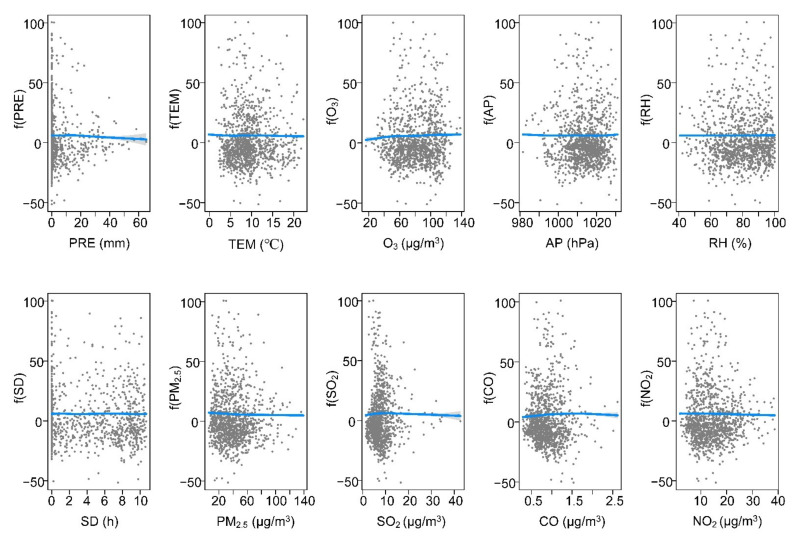
Exposure–response curves among meteorological variables, air pollution variables, and SARS-CoV-2 cases in MYR.

**Figure 6:**
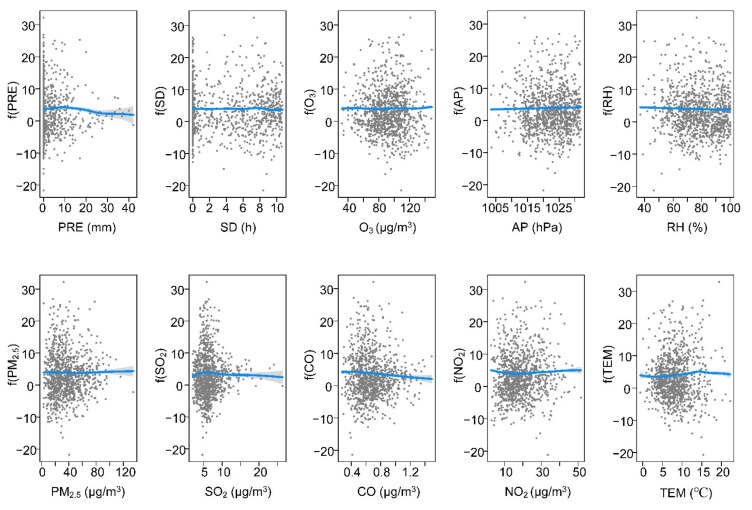
Exposure–response curves among meteorological variables, air pollution variables, and SARS-CoV-2 cases in YRD.

**Figure 7:**
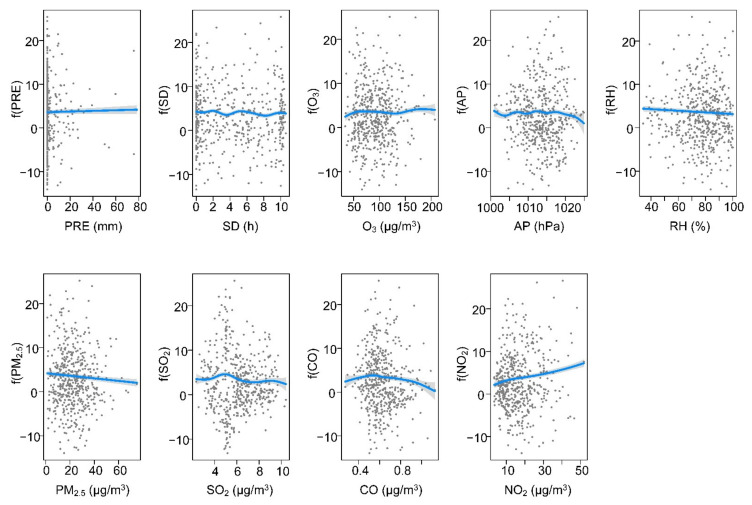
Exposure–response curves among meteorological variables, air pollution variables, and SARS-CoV-2 cases in PRD.

The exposure–response curves in Figure 4, Figure 5, Figure 6 and Figure 7 and the values of edf in Table 1 suggested that meteorological variables (PRE, TEM, AP, RH, and SD), air pollution variables (PM_2.5_, SO_2_, NO_2_, O_3_, and CO), and daily cumulative confirmed SARS-CoV-2 cases have either linear or nonlinear relationships. Specifically, in BTH, the relationship was approximately linear between SARS-CoV-2 and TEM (Figure 4 and Table 1). The edf value of PRE was close to 1, indicating that it has an approximately linear relationship with SARS-CoV-2. O_3_, CO, and NO_2_ were significantly positively linked with SARS-CoV-2. The SARS-CoV-2–air pressure curve initially showed a negative slope, but its trend changed when the air pressure was more than 970 hpa. In addition, the association between SARS-CoV-2 and RH and SO_2_ showed a negative direction. Although PRE, AP, and PM_2.5_ had a nonlinear relationship with SARS-CoV-2, they failed to pass the significance test (*p* > 0.05).

In the MYR, Table 1 showed that the relationship between each variable and SARS-CoV-2 was nonlinear (edf >1). Figure 5 showed that the changes in meteorological variables and air pollution variables have little impact on the propagation of SARS-CoV-2, which is consistent with the small interpretation rate of SARS-CoV-2 variations in the GAM model (35.2%). SARS-CoV-2 was significantly negatively linked with PRE, TEM, PM_2.5_, SO_2_, and NO_2_. The curve of each pair of relationships showed a relatively flatter shape. There was an inverse link between the SARS-CoV-2 and O_3_. In MYR, SD and RH had no significant effect on SARS-CoV-2 (*p* > 0.05).

In the YRD, the relationship between SARS-CoV-2 and AP was linear (edf = 1), while other variables had a nonlinear relationship with SARS-CoV-2 (edf >1). The association between SARS-CoV-2 and PRE was only positive when the precipitation was below 10 mm and negative in other cases (Figure 6). In the temperature range of 0–15 °C, the SARS-CoV-2 cases increased gradually, but in other cases, the SARS-CoV-2 cases decreased as the temperature increased. The curves between SARS-CoV-2, AP, and PM_2.5_ showed a positive slope. The curve between SARS-CoV-2 and SD seems to be a flat curve. The SARS-CoV-2 -NO_2_ curve showed a decreasing shape initially, but the curve changed positively when the NO_2_ concentration was between 15 and 50 μg/m^3^. Additionally, the curve was downward sloped between SARS-CoV-2 and RH, CO, and SO_2_. In YRD, O_3_ and PM_2.5_ had no significant effect on SARS-CoV-2 (*p* > 0.05).

In the PRD, the SARS-CoV-2 variations were linear with PRE, PM_2.5_, and RH (edf = 1) (Table 1). The association between SARS-CoV-2 and CO indicated a positive slope between 0 and 0.6 μg/m^3^, but showed an inverse trend in other cases. The curve between SARS-CoV-2 and PRE and NO_2_ appeared to be an increasing curve. The curves of SARS-CoV-2 and O_3_ showed a fluctuating upward trend, while the curves with AP and SO_2_ showed a fluctuating downward trend. SARS-CoV-2 showed a clear negative linkage with RH and PM_2.5_.

## 4. Discussion

### 4.1. Effects of Meteorological and Air Pollution Variables on SARS-CoV-2 in Urban Agglomerations

At present, the COVID-19 outbreak is still severe worldwide, bringing a huge medical and economic burden to many countries globally. It is worth recognizing that the spread of SARS-CoV-2 is affected by many variables. Previous studies have quantified the impact of meteorological and air pollution variables on SARS-CoV-2, but the results are inconsistent [7,8,13,23]. Available evidence demonstrates that air pollution and meteorological variables might negatively impact several physiological systems and organs of individuals of all ages [8,24]. A study carried out in seven metropolitan cities and nine provinces of Korea supported the link of SARS-CoV-2 cases with air pollution variables [24]. In this study, we assumed that meteorological and air pollution variables affected SARS-CoV-2, and different regions might cause different impact laws. Our results showed that meteorological and air pollution variables jointly affected SARS-CoV-2. In the four urban agglomerations, the increase in relative humidity and SO_2_ reduced the risk of SARS-CoV-2 transmission, while the curves of O_3_ and SARS-CoV-2 showed the opposite trend (Figure 4, Figure 5, Figure 6 and Figure 7). Some findings were consistent with a study in Lombardy, Italy [25]. The association between other variables and SARS-CoV-2 might show different trends with different urban agglomerations (Figure 4, Figure 5, Figure 6 and Figure 7, Table 1). In BTH, meteorological and air pollution variables could explain more than 75% of the SARS-CoV-2 variations (Table 1). Additionally, in YRD and PRD, multiple variables explained 45.6% and 62.2% of the SARS-CoV-2 variations, respectively, while the MYR value was only 35.2%. This result indicated that SARS-CoV-2 might have a complex transmission mechanism [8,26], and the research results in a single region were difficult to apply to other regions.

Analyzing the relationship between SARS-CoV-2 cases and the influencing factors, most studies rely on data from a certain region [2,18,27], which may be limited. In MYR and PRD, PM_2.5_ was negatively correlated with SARS-CoV-2, which was inconsistent with previous studies in Italian province capitals [28]. This might be because people take stricter protective measures when PM_2.5_ is higher, which reduces human-to-human transmission of SARS-CoV-2. For meteorological variables, in BTH, the relationship between SARS-CoV-2 and temperature was linear (Table 1 and Figure 4). However, in either MYR or YRD, the curve showed a nonlinear relationship (Figure 5 and Figure 6). Previous studies indicated that an elevated temperature was harmful to the virus [29,30]. Research also showed that temperature conditions were beneficial to the coronavirus [31,32]. There are some places where the epidemic is still severe despite hot weather, such as parts of India. In our study, precipitation and SARS-CoV-2 had different curves in urban agglomerations. However, the association between SARS-CoV-2 and precipitation in different studies also varies in time and space [8,33]. Therefore, the relationship between meteorological and air pollution variables and SARS-CoV-2 is complex and variable, and virus mutation and other variables may affect the relationship. Our results also indicated that the meteorological variables and the air pollution variables were significantly correlated in many cases (Figure 3). Previous studies have shown that meteorological variables can affect the diffusion of atmospheric pollutants [10,34], so the two are related. The relationship between variables may also contribute to the difficulty of clarifying the drivers of SARS-CoV-2.

In this study, the four urban agglomerations have different ecosystem, climatic, terrain, social, and economic characteristics, which may be the reason for the spatial heterogeneity of the relationship between the SARS-CoV-2 cases and influencing variables. A previous study indicated that the SARS-CoV-2 epidemic harmed mental health and verified the positive effects of the residential tree canopy on psychological distress in Beijing, China [22]. Additionally, Roviello and Roviello [15] concluded that the lowest SARS-CoV-2 impact in Italy in terms of mortality was observed in three Mediterranean regions endowed with high rates of forest area per capita, sustaining the hypothesis that the abundance of evergreen Mediterranean vegetation could have played a role in the milder outcome of the virus. From another perspective, these finds may also be caused by the health system. Furthermore, higher population densities have proven to contribute to the greater spread of SARS-CoV-2 in previous studies [16]. Other variables, such as pollen and ultraviolet radiation, have also been proven to be factors affecting SARS-CoV-2 transmission [20,35]. Therefore, in subsequent research, we may include more variables in the analysis.

### 4.2. Implications for the SARS-CoV-2 Control and Prevention

Cities in urban agglomerations often have some similarities in economic, social development, and geographical characteristics. This study takes urban agglomerations as the research object, which can better reflect the close connection between cities. It also aims to call on the government and the public to recognize this connection and strengthen research on epidemic prevention and control in urban agglomerations.

Even though the dispersal of SARS-CoV-2 cases is affected by many countermeasures and medical conditions, our results indicated that meteorological and air pollution variables were associated with SARS-CoV-2. The COVID-19 pandemic uncovers the fragility and weakness of our ecosystem, and lacks knowledge of controlling and preventing the pandemic [8]. In this study, the transmission of SARS-CoV-2 in different urban agglomerations was significantly correlated with a variety of factors, but the relationship was spatially differentiated. These findings indicated that SARS-CoV-2 prevention policies in different regions should be formulated according to local, meteorological, and air pollution conditions. Specifically, more attention should be given to variables that showed a significant trend in the relationship curve with SARS-CoV-2. From the exposure–response curves, when atmospheric pressure is above 980 hPa, relative humidity is low, and the content of CO and NO_2_ in the air is high, the awareness of SARS-CoV-2 transmission risk should be strengthened in BTH (Figure 4). Meteorological and air pollution variables had less impact on SARS-CoV-2 in MYR, so other measures are needed to combat the pandemic (Table 1 and Figure 5). In YRD, when there is drought and little rain, the atmospheric pressure is high and the temperature is 8–15 °C, the risk of epidemic transmission may increase, and more attention is needed (Figure 6). The PRD had a higher risk of SARS-CoV-2 transmission when relative humidity was low. As O_3_ and NO_2_ were the main air pollutants in PRD (Figure S2), SARS-CoV-2 prevention and control measures should be strengthened when the two values increase (Figure 7). Of course, it is not enough to rely on these alone. The government should continue to strictly implement SARS-CoV-2 prevention policies and adopt innovative, specialized, and advanced systems, including empowered moving and internet hospitals, as well as high technologies. Moreover, the establishment of a rational and efficient global health care system is essential not only for SARS-CoV-2, but also for unknown infections in the future [36].

### 4.3. Limitations of the Present Study

However, there are many limitations in the study. First, the air pollution data measured at the monitoring points were used for the study, which might lead to certain exposure measurement errors. Other variables, such as the use of air purifiers, the ventilation of the building, and the time and location of a person’s activities, can affect exposure levels [3,4,5]. These anthropogenic activities or measures are difficult to measure, and thus may have an impact on air pollution exposure, which in turn affects the results of the study. Second, the research time was selected from the first wave of COVID-19 in China, and the limited research time may reduce the statistical effect.

## 5. Conclusions

In this study, the impacts of meteorological and air pollution variables on SARS-CoV-2 cases were evaluated using a GAM analysis based on data from four urban agglomerations. The four urban agglomerations were all located in the Northern Hemisphere, and their meteorological variables had certain regional differentiation characteristics. Air pollution and meteorological variables tended to be strongly correlated. The nature of the relationship between SARS-CoV-2 and meteorological and air pollution variables (i.e., linear or nonlinear) varied with urban agglomerations. Four GAM models of urban agglomeration were constructed. The deviance explained by meteorological and air pollution variables on the SARS-CoV-2 variations was 75.4%, 35.2%, 45.6%, and 62.2% for BTH, MYR, YRD, and PRD, respectively. Our results showed that SARS-CoV-2 responses to meteorological and air pollution variables varied in different regions, making it difficult to use a single conclusion to support the formulation of epidemic prevention and control measures in all regions. Therefore, follow-up measures based on local meteorological and air pollution conditions may be a more effective approach to preventing and controlling SARS-CoV-2.

## Figures and Tables

**Table 1:** GAM model hypothesis test results of SARS-CoV-2 and multiple variables in four urban agglomerations.

		AP	RH	SD	PRE	TEM	SO_2_	CO	NO_2_	O_3_	PM_2.5_	Deviance Explained (%)
BTH	edf	5.08	8.24	5.99	1.78	1	7.14	5.65	5.83	5.07	5.53	75.4
	*p*	<0.001	<0.001	<0.001	0.06	0.08	<0.001	<0.01	<0.001	<0.001	0.07	
MYR	edf	5.34	1.68	6.02	3.81	4.74	4.5	4.27	2.26	5.33	3.18	35.2
	*p*	0.02	0.4	0.48	<0.01	0.04	<0.001	<0.001	<0.001	<0.001	<0.001	
YRD	edf	1	1.57	6.93	5.73	7.47	4.92	3.31	4.94	5.82	3.13	45.6
	*p*	<0.001	<0.01	<0.001	<0.001	<0.001	<0.001	<0.001	<0.001	0.17	0.3	
PRD	edf	8.4	1	8.27	1	-	6.59	5.48	4.58	6.49	1	62.2
	*p*	<0.001	<0.001	<0.001	0.15		<0.001	<0.001	<0.001	<0.001	<0.001	

Notes: edf, equivalent degrees of freedom.

## Data Availability

The data that support the findings of this study are available upon reasonable request from the authors.

## Supplementary Materials

The following supporting information can be downloaded at https://www.mdpi.com/article/10.3390/ijerph19010531/s1, Figure S1: Temporal variations of meteorological variables; Figure S2: Temporal variations of air pollution variables; Table S1: The cities involved in 4 main urban agglomerations in the study.
