# Supplementary material for: Assessment of Meteorological Variables and Air Pollution Affecting COVID-19 Cases in Urban Agglomerations: Evidence from China
Source: Int J Environ Res Public Health. 2022 Jan 4;19(1):531. doi: 10.3390/ijerph19010531 (PMC8744893; doi:10.3390/ijerph19010531)
Supplement: Supplementary file 1 [file ijerph-19-00531-s001.zip › Supplementary materials.pdf]

Assessment of meteorological variables and air pollution affecting COVID-19 cases in urban agglomerations: evidence from China

Mingyue Zhao [1], Yuanxin Liu [2,*] and Amatus Gyilbag [1]

[1] Institute of Environment and Sustainable Development in Agriculture, Chinese Academy of Agricultural Sciences, Beijing 100081, China
[2] Academy for Multidisciplinary Studies, Capital Normal University, Beijing 100048, China
[*] Corresponding at: yuanxinliu@cnu.edu.cn

Table S1 The cities involved in the 4 main urban agglomerations in the study.

No.	Name	Provinces involved	Cities involved
1	MYR	Hubei	Huangshi, Ezhou, Huanggang, Xiaogan, Xianning, Xiantao, Qianjiang, Tianmen, Xiangyang, Yichang, Jingzhou, Jingmen
		Hunan	Changsha, Zhuzhou, Xiangtan, Yueyang, Yiyang, Changde, Hengyang, Loudi
		Jiangxi	Nanchang, Jiujiang, Jingdezhen, Yingtan, Xinyu, Yichun, Pingxiang, Shangrao, Fuzhou, Ji'an
2	BTH	-	Beijing;
		-	Tianjin;
		Hebei	Baoding, Tangshan, Langfang, Shijiazhuang, Qinhuangdao, Handan, Xingtai, Zhangjiakou, Chengde, Cangzhou, Hengshui
3	YRD	-	Shanghai;
		Jiangsu	Nanjing, Wuxi, Changzhou, Suzhou, Nantong, Yancheng, Yangzhou, Zhenjiang, Taizhou
		Zhejiang	Hangzhou, Ningbo, Shaoxing, Huzhou, Jiaxing, Jinhua, Zhoushan, Taizhou, Wenzhou
		Anhui	Hefei, Wuhu, Maanshan, Tongling, Anqing, Xuancheng, Chizhou, Chuzhou
4	PRD	Guangdong	Guangzhou, Shenzhen, Foshan, Dongguan, Huizhou, Zhongshan, Zhuhai, Jiangmen, Zhaoqing, Shanwei, Yangjiang, Qingyuan, Yunfu, Heyuan

Abbreviations: BTH, Beijing-Tianjin-Hebei urban agglomeration; YRD, Yangtze River Delta urban agglomeration; MYR, Middle reaches of the Yangtze River urban agglomeration; PRD, Pearl River Delta urban agglomeration.

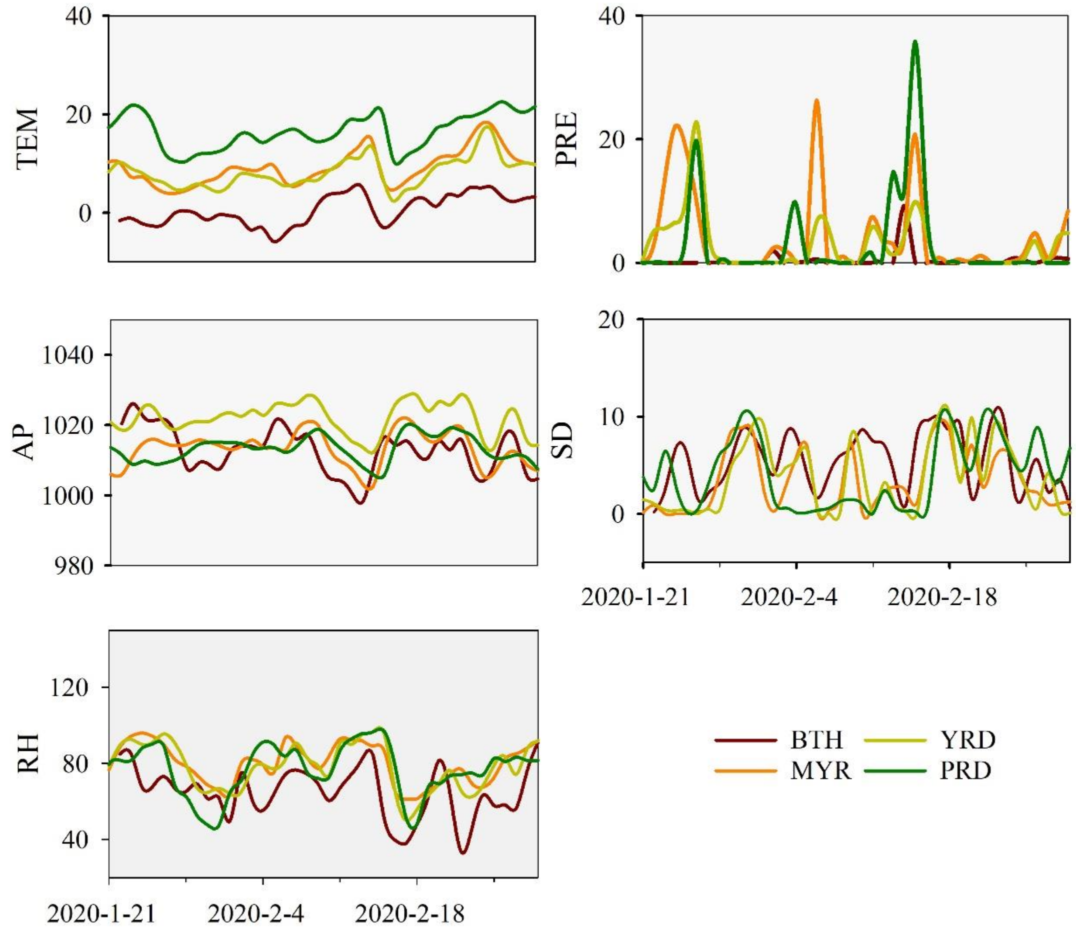

Figure S1 Temporal variations of meteorological variables

Abbreviations: TEM, temperature; PRE, precipitation; AP, atmospheric pressure; SD, sunshine duration; RH, relative humidity.

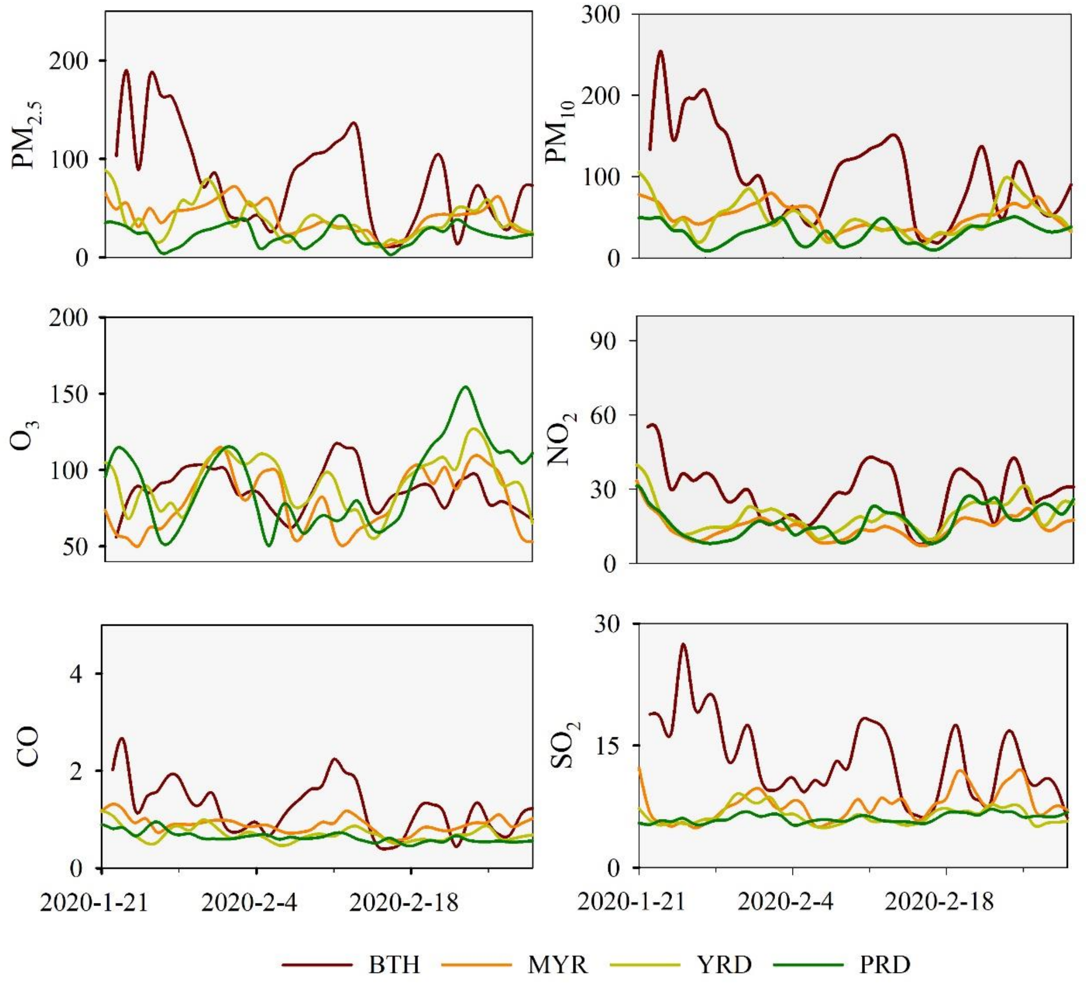

Figure S2 Temporal variations of air pollution variables

Abbreviations: $PM_{2.5}$, particulate matter with aerodynamic diameter ≤ 2.5μm ; PM_{10}, particulate matter with aerodynamic diameter ≤ 10μm ; O3, ozone; NO_2, nitrogen dioxide; CO, carbon monoxide; SO_2, sulfur dioxide.
